# Post-term growth and cognitive development at 5 years of age in preterm children: Evidence from a prospective population-based cohort

**DOI:** 10.1371/journal.pone.0174645

**Published:** 2017-03-28

**Authors:** Laure Simon, Simon Nusinovici, Cyril Flamant, Bertrand Cariou, Valérie Rouger, Géraldine Gascoin, Dominique Darmaun, Jean-Christophe Rozé, Matthieu Hanf

**Affiliations:** 1 Department of Paediatric Medicine, Nantes University Hospital, Nantes, France; 2 INSERM CIC 1413, Clinical Investigation Center, Nantes University Hospital, Nantes, France; 3 Department of Endocrinology, l’Institut du Thorax, Nantes University Hospital, Nantes, France; 4 Réseau “Grandir ensemble”, Nantes University Hospital, Nantes, France; 5 Department of Neonatal Medicine, Angers University Hospital, Angers, France; 6 National Institute for Agricultural Research, UMR 1280 PHAN, Nantes University, Institut des Maladies de l’Appareil Digestif (IMAD), and CRNH-Ouest, Nantes, France; 7 INSERM UMR 1181 Biostatistics, Biomathematics, Pharmacoepidemiology and Infectious Diseases (B2PHI), Versailles Saint Quentin University, Villejuif, France; Universidade de Sao Paulo, BRAZIL

## Abstract

While the effects of growth from birth to expected term on the subsequent development of preterm children has attracted plentiful attention, less is known about the effects of post-term growth. We aimed to delineate distinct patterns of post-term growth and to determine their association with the cognitive development of preterm children. Data from a prospective population-based cohort of 3,850 surviving infants born at less than 35 weeks of gestational age were used. Growth was assessed as the Body Mass Index (BMI) Z-scores at 3, 9, 18, 24, 36, 48, and 60 months. Cognitive development at five years of age was evaluated by the Global School Adaptation score (GSA). Latent class analysis was implemented to identify distinct growth patterns and logistic regressions based on propensity matching were used to evaluate the relationship between identified growth trajectories and cognitive development. Four patterns of post-term growth were identified: a normal group with a Z-score consistently around zero during childhood (n = 2,469; 64%); a group with an early rapid rise in the BMI Z-score, but only up to 2 years of age (n = 195; 5%); a group with a slow yet steady rise in the BMI Z-score during childhood (n = 510; 13%); and a group with a negative Z-score growth until 3 years of age (n = 676; 18%). The group with a slow yet steady rise in the BMI Z-score was significantly associated with low GSA scores. Our findings indicate heterogeneous post-term growth of preterm children, with potential for association with their cognitive development.

## Introduction

Among potential sequelae associated with preterm birth, sub-optimal cognitive development is prominent even in the absence of neonatal morbidities [1,2] with however a high individual variability among preterm children. To explain this observed heterogeneity, several studies have specifically focused in the past decade on the association of atypical growth during childhood with subsequent cognitive development [3–5]. Although considerable attention has been focused on the effects of growth from birth to expected term, less is known about the effects of post-term growth on the cognitive development of preterm children [6]. Because post-term growth is a modifiable risk factor, the associated long-term health consequences are of utmost significance to the management of preterm children in their follow-up care after discharge.

Furthermore, to improve the identification of existing relationships between growth and health outcomes, several recent longitudinal studies have tried to identify and characterize distinct trajectories of Body Mass Index (BMI) in pediatric population [7–14] using data-driven statistical methods, such as latent class analysis (LCA) [15–19]. Although these methods have the potential to identify subgroups of high-risk children and thus provide new strategies for early prevention or intervention, we found no study which used neither longitudinal data nor examine the possibility of individual heterogeneity in post-term growth of preterm children.

In this context, the purpose of the current study was 1) to delineate distinct patterns of post-term growth measured as BMI Z-score trajectories over a period of 5 years from a large prospective population-based cohort of preterm children, 2) to identify perinatal characteristics predictive of these BMI Z-score trajectories and 3) to determine whether BMI Z-score trajectories are associated with the cognitive development at 5 years of age.

## Methods

### Ethics statement

This observational study was performed in accordance with the French regulations. The LIFT cohort is indeed registered with the French data protection authority in clinical research (“Commission Nationale de l’Informatique et des Libertés”, No. 851117). This study was approved by a relevant ethic committee (groupe nantais d'éthique dans le domaine de la santé). Informed written consent was obtained from the two parents of each child prior to their inclusion.

### Study area and population

This study included all surviving infants born at less than 35 weeks of gestational age between 2003 and 2008 in the Pays de la Loire (PDL) region of France. The participants were enrolled in the regional Loire Infant Follow-up Team (LIFT) network, which was implemented in 2003 to follow infants born in the PDL region at a gestational age of 35 weeks or less. The LIFT network includes all the 24 maternity clinics located in the PDL, of which three have neonatal intensive care units. Trained physicians monitor the children in a standardized manner at 3, 9, 18, and 24 months and at 3, 4, and 5 years of age. In our analysis, we included children who had three or more measurements of their height and weight recorded as part of their medical follow-up to allow BMI trajectories to be reasonably inferred [16].

### Data collection

In the LIFT cohort, a large set of perinatal data are collected at birth and at hospital discharge for all included children. These data are extracted from medical records and mainly concern (i) mother administrative and socio-economic conditions (ii) pregnancy complications, (iii) medication during pregnancy, (iv) characteristics of the delivery (v) child's condition at birth and at discharge, (vi) neonatal diseases, (vii) organization of care and management after birth, (viii) child’s treatment and medication and (ix) brain anomalies assessed by ultrasound/magnetic resonance imaging techniques. Data concerning the growth, neurological, motor, visual and hearing assessment are also directly recorded by the neonatologists during the clinical examination preceding discharge.

A set of 20 exploratory variables describing perinatal characteristics shown or strongly suspected to be associated with the cognitive development and /or the post-term growth of children were specifically used in this study: 1) characteristics of the child (e.g. gender; gestational age estimated during the prenatal period using maternal dates of expected delivery based on last menstrual period, Z-scores of weight and head circumference at birth and at discharge computed according to the Olsen standards [20]; and the year of birth), 2) characteristics of the mother and her pregnancy (e.g. administration of antenatal corticosteroids, multiple pregnancy, hypertension during pregnancy, social security benefits for individuals with a low income, and socioeconomic level), 3) characteristics of the neonatal hospitalization (e.g. the apgar score, intubation at birth, late-onset infection defined as proven or highly probable bacterial infection occurring after the first three days of life and treated by antibiotics for 8 or more days, surgery, severe cranial ultrasound/MRI abnormalities defined as intraventricular hemorrhage with ventricular dilation and intraparenchymal hemorrhage, bronchopulmonary dysplasia defined as a requirement for supplemental oxygen at 28 days, and necrotizing enterocolitis), and 4) child nutrition/growth characteristics during hospitalization (e.g. the length of parenteral nutrition, breastfeeding defined as readily taking the breast at discharge, and the difference in Z-scores of birth weight and weight at discharge).

### BMI assessment

Post-term growth was assessed from 3 months to 5 years of age, using the BMI at 3, 9, 18, and 24 months and 3, 4, and 5 years of post-menstrual age. Weight, height, and head circumference (HC) were measured, either by a physician or other health personnel. Recumbent length was measured in children <2 years of age, and standing height was measured from the age of 2 onward. As recommended elsewhere [21], the post-term BMI as well as weight, height, and HC were transformed into standardized Z-scores by using the LMS method according to the ‘‘WHO child growth standards” for children less than 1,856 days of age, and according to the ‘‘WHO growth standards for school-aged children and adolescents” thereafter [22,23]. When studying growth in preterm children, it is additionally recommended to adjust for gestational age [21]. Mainly for practical reasons, it is also recommended to adjust for at least 2 years of chronological age [21]. Because this study is focused on the temporal dynamic of BMI-Z-score over a period going beyond 3 years of age, it was decided to adjust for gestational age on all growth measures (3 months – 5 years of age) in view to avoid artificial breaks in identified growth trajectories.

### Cognitive assessment at five years of age

The Global School Adaptation (GSA) score was used to assess the level of cognitive development of the preterm children at 5 years of age. It was originally designed as a tool for teachers to assess a child’s abilities and behavior in the classroom [24,25]. Our team has established that this score is a simple and reliable screening tool for assessing cognitive development in preterm children at five years of age [26]. In this validation study, the overall correlation between IQ and GSA scores was indeed 0.56 (0.50 when only considering children without severe brain anomalies) and only 10.8% of children were shown to have divergent IQ and GSA scores. At the age of five years ± two months, the questionnaire was given to the parents of the children followed by the network, who then forwarded it to the children’s teachers. It comprised six questions investigating linguistic competence, and five questions investigating non-verbal abilities. A further eight questions addressed the child’s behavior in the classroom. The final question solicited the teacher’s outlook in regard to the child’s future adaptation to school life. The total score was calculated by adding the points from the 20 questions, thus resulting in values ranging from 20 to 60.

### Statistical analysis

All of the analyses were performed with the statistical software R. LCA identifies distinct groups of individuals who are homogeneous with respect to the kinetics of change of a factor over time, but heterogeneous as compared with other groups [27]. The LCA model was specified as a linear mixed-effects model with BMI Z-scores as the dependent variable. A mixed-effect model was used to account for the correlation of repeated measurements with each child. The trajectory shape was modeled using a cubic function of BMI assessment times. To identify the number of trajectories, we selected the model having the lowest Bayesian Information Criterion (BIC). To warrant clinical relevance, however, we used a prior requirement of at least 5% of the population of preterm children in each group. A posterior probability of membership in each of the identified latent trajectories was calculated for each child. Each child was then assigned exclusively to the trajectory for which the highest probability was obtained. To determine the perinatal factors predicting the identified BMI Z-score trajectory memberships, a multiple multinomial logistic regression, weighted by the estimated posterior trajectory membership probabilities, was computed. The set of 20 previously described exploratory variables were simultaneously used in this multiple analysis. The “normal” trajectory was defined as the reference trajectory. To evaluate the relationship between abnormal trajectories and low GSA scores, GSA scores were dichotomized according to the 25th percentile (GSA scores <44, versus GSA scores >44). Similarly to recent studies in the area [28], models based on propensity score matching were used to avoid bias due to systematic differences in the distribution of baseline characteristics between exposed and non exposed children and balance confounders between exposure groups, thus reducing bias [29]. Because gestational age, birth weight Z-score, and gender are known to be strongly associated with the cognitive development of children, we supplementary adjusted final models with these three variables. To assess the robustness of our results, crude associations as well as associations adjusted only on gestational age, birth weight Z-score and gender were also computed. To calculate the propensity scores, predictions from the previously described multinomial logistic regression model were used. Calculated propensity scores were thus based on the set of 20 previously described exploratory variables. For each abnormal trajectory, we used a 1:3 matching algorithm without replacement, to match children belonging to this trajectory with children belonging to the “normal” trajectory based on gestational age, gender, birth weight Z-score, and propensity score within a caliper of 0.2 standard deviations of the logit of the propensity score [30]. Imbalance after matching was checked. Logistic regressions fit by generalized estimating equations were used to account for paired data [31].

## Results

**Fig 1:**
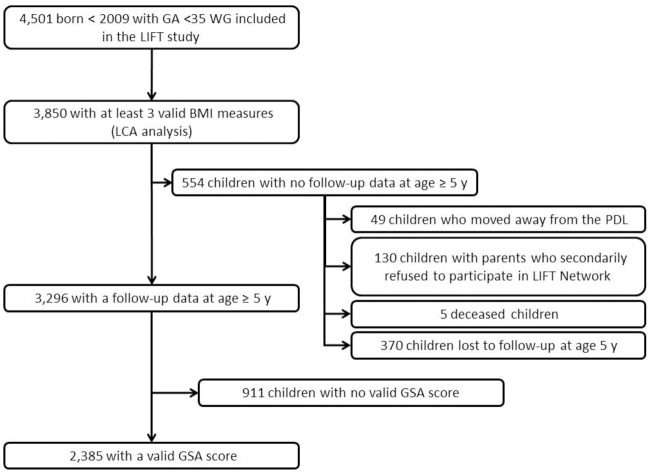
Study flow chart.

4,501 children born at less than 35 weeks of gestational age between March 2003 and December 2008 were included in the LIFT network (Fig 1).

Descriptive statistics of children included and not included in the LCA analysis are presented in Table 1. Children not included differed significantly in terms of gender, gestational age, socioeconomic level, multiple pregnancy, administration of antenatal corticotherapy, severe cranial abnormalities, late-onset infection occurrence, length of parenteral nutrition, and breastfeeding at discharge. Of the 3,850 children included in the LCA analysis, BMI measurements at 3, 9, 18, 24 months and 3, 4, and 5 years of age were available for 3,516 (91%); 3,486 (91%); 3,429 (89%); 3,413 (89%); 3,107 (81%); 2,605 (68%); and 2,148 (56%) of the participants, respectively.

**Table 1:** Comparison of children included and not included in the LCA analysis.

Variable	Category	Children not included in the LCA (N = 651)	Children included in the LCA (N = 3, 850)	P value*
***Children’s characteristics***				
**Child’s gender, n (%)**	*Male*	343 (52.7)	2, 086 (54.2)	<0.001
**Gestational age, n (%)**	*22–29*	105 (16.1)	746 (19.4)	0.009
	*30–31*	121 (18.6)	716 (18.6)	
	*32–33*	217 (33.3)	1387 (36)	
	*34–35*	208 (32.0)	1001 (26)	
weight Z-score at birth**	*Median (IQR)*	-0.2 (-0.9, 0.4)	-0.3 (-1, 0.4)	0.247
HC Z-score at birth**	*Median (IQR)*	-0.1 (-0.7, 0.5)	-0.1 (-0.7, 0.5)	0.903
weight Z-score at discharge**	*Median (IQR)*	-0.9 (-1.5, -0.3)	-0.9 (-1.6, -0.3)	0.366
height Z-score at discharge**	*Median (IQR)*	-1.2 (-1.9, -0.6)	-1.2 (-1.9, -0.5)	0.891
HC Z-score at discharge**	*Median (IQR)*	-0.2 (-0.7, 0.3)	-0.2 (-0.7, 0.4)	0.387
**Year of birth, n (%)**	2003–2004	179 (27.5)	1, 101 (28.6)	0.427
	2005–2006	214 (32.9)	1, 326 (34.4)	
	2007–2008	258 (39.6)	1, 423 (37.0)	
***Mother and pregnancy characteristics***				
**Socioeconomic level, n (%)**	*Higher level*	47 (7.2)	1, 000 (26)	< 0.001
**Social security benefits for low income, n (%)**	Yes	58 (8.9)	420 (10.9)	0.144
**Multiple pregnancy, n (%)**	Yes	190 (29.2)	1, 456 (37.8)	< 0.001
**Antenatal corticotherapy, n (%)**	Yes	306 (47.0)	2, 094 (54.4)	< 0.001
**Hypertension during pregnancy, n (%)**	Yes	106 (16.3)	546 (14.2)	0.178
***Neonatal hospitalization characteristics***				
**Surgery, n (%)**	Yes	17 (2.6)	76 (2.0)	0.364
**Severe cranial ultrasound/MRI abnormalities, n (%)**	Yes	51 (7.8)	139 (3.6)	< 0.001
**Intubation at birth, n (%)**	Yes	103 (15.8)	620 (16.1)	0.902
**Apgar at 5 min, n (%)**	<7	35 (5.4)	180 (4.7)	0.499
**Late-onset infection, n (%)**	Yes	67 (10.3)	529 (13.7)	0.019
**Necrotizing enterocolitis, n (%)**	Yes	6 (0.9)	22 (0.6)	0.434
**Bronchopulmonary dysplasia, n (%)**	No oxygen therapy	443 (68.0)	2, 525 (65.6)	0.260
	O2<28 days	180 (27.6)	1, 106 (28.7)	
	O2 ≥ 28 days	28 (4.3)	219 (5.7)	
***Child nutrition/growth characteristics during hospitalization***				
Delta weight Z-scores**	*Median (IQR)*	-0.6 (-1, -0.2)	-0.6 (-1.1, -0.2)	0.957
**Length of parenteral nutrition, n (%)**	No	221 (33.9)	1, 054 (27.4)	< 0.001
	< 11 days	296 (45.5)	1, 725 (44.8)	
	≥ 11 days	134 (20.6)	1, 071 (27.8)	
**Breastfeeding at discharge, n (%)**	Yes	81 (12.4)	659 (17.1)	0.004

IQR: interquartile range; BMI: Body Mass Index; HC: Head Circumference

* Categorical variables were compared using χ2 test and continuous ones with Wilcoxon test

** Z-scores were computed according to Olsen’ standards

### BMI Z-score trajectories

**Fig 2:**
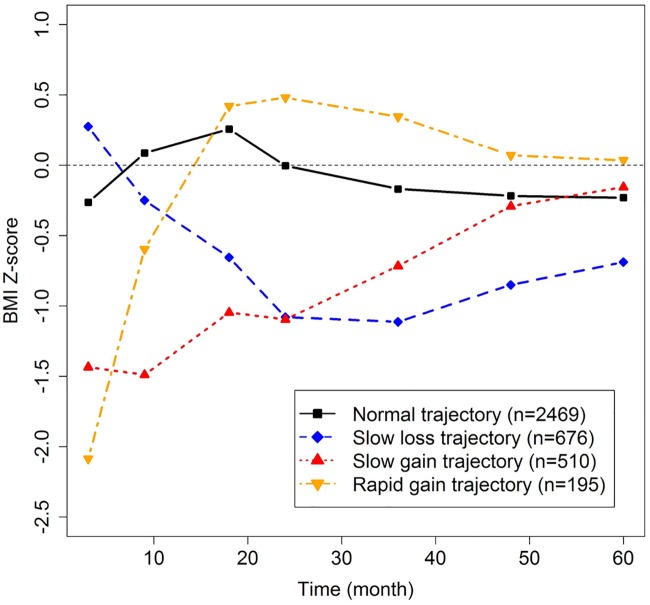
Longitudinal BMI Z-score trajectories identified by the latent class modeling. Z-scores were computed according to the ‘‘WHO child growth standards” for children less than 1,856 days of age, and according to the ‘‘WHO growth standards for school-aged children and adolescents” thereafter.

The four distinct mean trajectories that best characterized the complex developmental course of BMI Z-scores over the first 5 years of life in the included preterm children are shown in Fig 2. The associated developmental course of weight, height, and HC Z-scores were also plotted (Figure A in S1 File). The same trajectories were observed when only one child from those of a same multiple pregnancy was included in the LCA (Figure A in S1 File).

Descriptive statistics of children according to each trajectory are presented in Tables A and B in S1 File. The “normal” trajectory was the most common (n = 2,469; 64%), and was characterized by a mean value that was more or less a flat line deviating by approximately -0.25 Z-score from the null line for ideal growth as defined by the WHO standards. The ‘‘slow loss” trajectory, comprising 18% (n = 676) of the children, had an initial positive mean Z-score level at 3 months of age, and a negative Z-score growth until 3 years of age, which gradually became less negative thereafter. The ‘‘slow gain” trajectory, comprising about 13% (n = 510) of the children, had an initial negative mean Z-score level at 3 months of age, with a subsequent continuous rise in the BMI Z-score over the 5 year period. The ‘‘rapid gain” trajectory, comprising only about 5% (n = 195) of the children, had a low initial mean Z-score level at 3 months of age, and an early rapid rise in the Z-score up to 2 years of age only, followed by a slow decline thereafter. At five years of age, the mean Z-score levels of the four trajectories were close to the WHO standards.

### Perinatal predictors of BMI Z-score trajectories

Associations of perinatal predictors with BMI Z-score trajectories are presented in Table 2. The “normal” trajectory was taken as the reference group. Male children with a gestational age of less than 32 weeks, a birth HC Z-score less than -1, a difference in Z-scores between birth weight and weight at discharge exceeding -1, or who were not being breast fed at the time of discharge, had a significantly higher risk of fitting the “slow loss” trajectory (p<0.05). Male children with a negative birth weight Z-score had a significantly higher risk of fitting the “slow gain” trajectory, compared to the “normal” trajectory. Male children, having had a late-onset infection during their hospitalization, a difference in Z-scores between birth weight and weight at discharge of less than -1, or who were being breast fed at the time of discharge, had a significantly higher risk of fitting the “rapid gain” trajectory.

**Table 2:** Results from the multiple multinomial logistic regression. The reference group is the “normal” trajectory.

Variable	Category	Slow loss trajectory (N = 676) aOR [95% CI]	Slow gain trajectory (N = 510) aOR [95% CI]	Rapid gain trajectory (N = 195) aOR [95% CI]
***Children’s characteristics***				
**Child’s gender** *(Ref*: *Female)*	*Male*	1.35 [1.09, 1.66]*	1.56 [1.22, 1.99]*	1.62 [1.14, 2.29]*
**Gestational age** *(Ref*: *>33)*	22–29	1.64 [1.07, 2.52]*	1.58 [0.97, 2.55]	0.53 [0.26, 1.09]
	30–31	1.63 [1.16, 2.29]*	1.31 [0.88, 1.95]	0.77 [0.43, 1.36]
	32–33	1.18 [0.89, 1.57]	1.11 [0.80, 1.54]	0.92 [0.59, 1.45]
**Birth weight Z-score** *(Ref*: > 0)**	< -1	1.16 [0.81, 1.64]	3.23 [2.18, 4.79]*	1.51 [0.85, 2.68]
	[-1; 0[	1.16 [0.89, 1.50]	1.50 [1.09, 2.08]*	0.96 [0.62, 1.48]
**Birth HC Z-score** *(Ref*: > 0)**	< -1	1.54 [1.06, 2.24]*	1.16 [0.76, 1.76]	0.87 [0.46, 1.63]
	[-1; 0[	1.28 [0.99, 1.67]	1.03 [0.75, 1.42]	0.78 [0.50, 1.21]
	Not known	1.23 [0.83, 1.81]	1.17 [0.74, 1.85]	0.71 [0.35, 1.43]
**Year of birth** *(Ref*: *2003–2004)*	2005–2006	0.92 [0.71, 1.20]	0.82 [0.61, 1.11]	0.80 [0.52, 1.24]
	2007–2008	0.98 [0.76, 1.27]	0.92 [0.68, 1.24]	0.96 [0.63, 1.46]
***Mother and pregnancy characteristics***				
**Socioeconomic level** *(Ref*: *Lower level)*	*Higher level*	0.78 [0.61, 1.01]	0.92 [0.70, 1.21]	0.90 [0.61, 1.32]
**Social security benefits for low income** *(Ref*: *No)*	Yes	0.75 [0.53, 1.06]	0.84 [0.57, 1.24]	0.99 [0.58, 1.69]
**Multiple pregnancy** *(Ref*: *No)*	Yes	1.10 [0.88, 1.37]	1.14 [0.89, 1.46]	0.90 [0.62, 1.30]
**Antenatal corticotherapy** *(Ref*: *No)*	Yes	0.91 [0.73, 1.12]	1.01 [0.79, 1.30]	1.06 [0.75, 1.50]
**Hypertension during pregnancy** *(Ref*: *No)*	Yes	0.98 [0.73, 1.32]	1.04 [0.74, 1.46]	1.21 [0.76, 1.93]
***Neonatal hospitalization characteristics***				
**Surgery** *(Ref*: *No)*	Yes	1.57 [0.71, 3.44]	1.68 [0.77, 3.66]	1.32 [0.46, 3.74]
**Severe cranial ultrasound/MRI abnormalities** *(Ref*: *No)*	Yes	1.19 [0.69, 2.04]	0.98 [0.53, 1.82]	1.05 [0.46, 2.38]
**Intubation at birth** *(Ref*: *No)*	Yes	1.12 [0.79, 1.59]	0.83 [0.55, 1.24]	1.67 [0.96, 2.89]
**Apgar score at 5 min** *(Ref*: *≥7)*	<7	0.97 [0.58, 1.65]	1.31 [0.75, 2.27]	1.26 [0.62, 2.59]
**Late-onset infection** *(Ref*: *No)*	Yes	0.81 [0.56, 1.16]	0.97 [0.67, 1.42]	1.72 [1.02, 2.91]*
**Necrotizing enterocolitis** *(Ref*: *No)*	Yes	0.46 [0.07, 3.21]	1.33 [0.35, 5.05]	1.66 [0.37, 7.48]
**Bronchopulmonary dysplasia** *(Ref*: *No oxygen therapy)*	O2<28 days	1.03 [0.80, 1.31]	1.06 [0.79, 1.41]	1.05 [0.70, 1.58]
	O2 ≥ 28 days	0.83 [0.48, 1.44]	1.29 [0.76, 2.18]	1.49 [0.71, 3.12]
***Child nutrition/growth characteristics during hospitalization***				
**Delta weight Z-score** *(Ref*: >0)**	]-1; 0]	0.78 [0.59, 1.04]	1.13 [0.79, 1.60]	1.35 [0.74, 2.47]
	<-1	0.65 [0.45, 0.93]*	1.34 [0.88, 2.05]	2.15 [1.11, 4.16]*
	Not known	0.74 [0.51, 1.07]	0.94 [0.59, 1.48]	1.93 [0.97, 3.84]
**Length of parenteral nutrition** *(Ref*: *No)*	< 11 days	0.94 [0.72, 1.22]	0.82 [0.60, 1.12]	0.73 [0.48, 1.12]
	≥ 11 days	0.93 [0.66, 1.29]	0.91 [0.63, 1.32]	0.71 [0.41, 1.21]
**Breastfeeding at discharge** *(Ref*: *No)*	Yes	0.71 [0.52, 0.96]*	0.98 [0.71, 1.35]	1.57 [1.04, 2.37]*

All the variables presented in Table 2 were incorporated in the multiple model.

BMI: body Mass Index; aOR: Adjusted odds ratio; CI: Confident interval; HC: Head Circumference

* p<0.05

** Z-scores were computed according to Olsen’ standards

### Association between GSA score and BMI Z-score trajectories

On the 3,850 children included in the latent class analysis, only 2,385 (62%) children had no missing data in regard to the GSA score. Descriptive statistics of children with or without GSA scores are presented in Table 3. Children with no GSA score differed significantly in terms of year of birth, socioeconomic level, social security benefits for low income and breast feeding at discharge.

**Table 3:** Comparison of preterm children included and not included in the GSA analysis.

Variable	Category	Children with GSA score (N = 2385)	Children without GSA score (N = 1,465)	P value*
***Children’s characteristics***				
**Child’s gender**	*Male*	1,297 (54.4)	789 (53.9)	0.776
**Gestational age**	*22–29*	463 (19.4)	283 (19.3)	0.621
	*30–31*	431 (18.1)	285 (19.5)	
	*32–33*	875 (36.7)	512 (34.9)	
	*34–35*	616 (25.8)	385 (26.3)	
Birth weight Z-score**	*Median (IQR)*	-0.2 (-1.0,0.4)	-0.3 (-1.0,0.3)	0.465
Birth HC Z-score**	*Median (IQR)*	-0.1 (-0.7,0.6)	-0.2 (-0.8,0.5)	0.215
Discharge weight Z-score**	*Median (IQR)*	-0.9 (-1.6,-0.4)	-1.0 (-1.6,-0.3)	0.858
Discharge height Z-score**	*Median (IQR)*	-1.2 (-1.9,-0.5)	-1.2 (-1.9,-0.5)	0.301
Discharge HC Z-score**	*Median (IQR)*	-0.2 (-0.7,0.4)	-0.2 (-0.7,0.4)	0.268
**Year of birth**	2003–2004	681 (28.6)	420 (28.7)	
	2005–2006	789 (33.1)	537 (36.7)	0.035
	2007–2008	915 (38.4)	508 (34.7)	
***Mother and pregnancy characteristics***				
**Socioeconomic level**	*Higher level*	689 (28.9)	311 (21.2)	< 0.001
**Social security benefits for low income**	Yes	229 (9.6)	191 (13)	0.001
**Multiple pregnancy**	Yes	908 (38.1)	548 (37.4)	0.705
**Antenatal corticotherapy**	Yes	1297 (54.4)	797 (54.4)	0.999
**Hypertension during pregnancy**	Yes	338 (14.2)	208 (14.2)	0.999
***Neonatal hospitalization characteristics***				
**Surgery**	Yes	49 (2.1)	27 (1.8)	0.735
**Severe cranial ultrasound/MRI abnormalities**	Yes	83 (3.5)	56 (3.8)	0.643
**Intubation at birth**	Yes	386 (16.2)	234 (16)	0.898
**Apgar at 5 min**	<7	111 (4.7)	69 (4.7)	0.999
**Late-onset infection**	Yes	324 (13.6)	205 (14)	0.757
**Necrotizing enterocolitis**	Yes	11 (0.5)	11 (0.8)	0.880
**Bronchopulmonary dysplasia**	No oxygen therapy	1,563 (65.5)	962 (65.7)	0.757
	O2<28 d	691 (29)	415 (28.3)	
	O2 ≥ 28 d	131 (5.5)	88 (6)	
***Child nutrition/growth characteristics during hospitalization***				
Delta weight Z-score**	*Median (IQR)*	-0.6 (-1.1, -0.2)	-0.6 (-1.1, -0.1)	0.408
**Length of parenteral nutrition**	No	660 (27.7)	394 (26.9)	0.850
	< 11 days	1067 (44.7)	658 (44.9)	
	≥ 11 days	658 (27.6)	413 (28.2)	
**Breast feeding at discharge**	Yes	446 (18.7)	213 (14.5)	0.001

IQR: interquartile range; BMI: Body Mass Index; HC: Head Circumference

* Categorical variables were compared using χ2 test and continuous ones with Wilcoxon test

** Z-scores were computed according to Olsen’ standards

**Fig 3:**
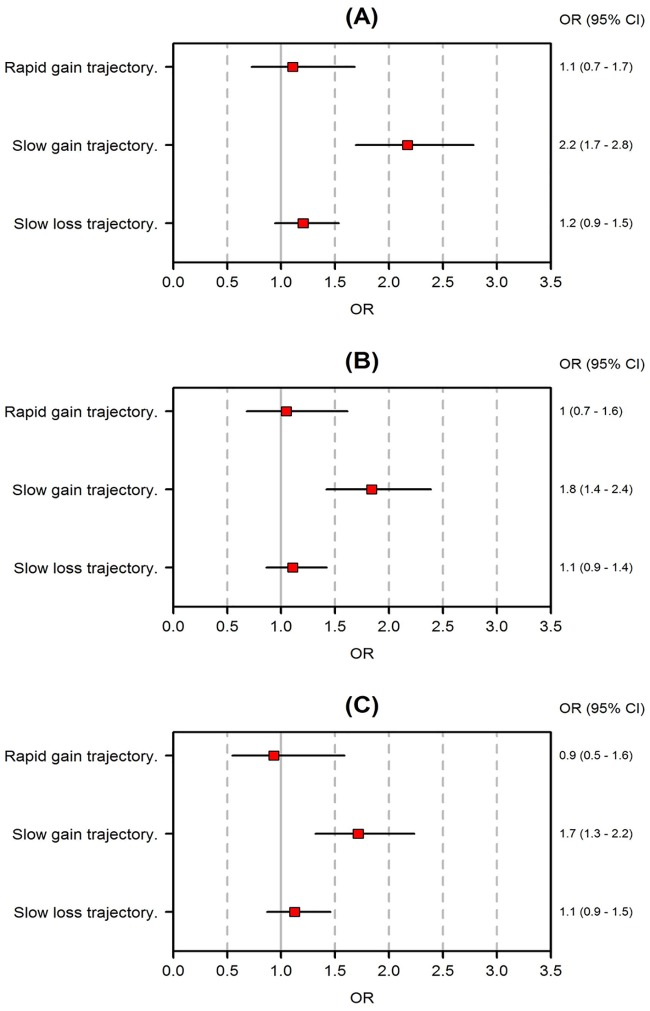
Association between abnormal BMI Z-score trajectories and low GSA scores at five years of age using (A) unadjusted logistic regression (B) logistic regression adjusted for gender, birth weight Z-score, and gestational age and (C) logistic regressions adjusted for gender, birth weight Z-score, and gestational age based on propensity matching. The reference trajectory is the “normal” trajectory. Propensity score matching were performed using a set of 20 variables reflecting the characteristics of the child, the characteristics of the mother and her pregnancy, the characteristics of the neonatal hospitalization, as well as the child nutrition/growth characteristics during hospitalization.

The median GSA score was 52 in children with GSA score [interquartile range: 44–56]. Fig 3 shows the associations of BMI Z-score trajectories with GSA scores < 44. In the three models, only the “slow gain” trajectory was significantly and positively associated with low GSA scores at five years of age, when compared to the “normal” trajectory. Neither the inclusion in the analysis of only one child from those of a same multiple pregnancy (Figure C in S1 File) nor a supplementary adjustment based on hospital growth (Figure D in S1 File) modified the observed relation. The matched groups were found to be well balanced for all of the recorded variables (Tables E, F and G in S1 File).

## Discussion

In this study, four distinct groups of post-term BMI Z-score trajectories from 3 months to 5 years of age were identified in preterm children by the LCA analysis. Overall, the BMI Z-scores were very similar at 60 months of age in the four identified trajectories. Confirmation of these results would underscore the appropriateness of focusing on the temporal dynamics of post-term growth to study its association with development of preterm children.

Children from the three “abnormal” trajectories differed significantly in regard to important variables, such as the children’s gender, gestational ages, birth weights, and HC Z-scores, hospital growth and breastfeeding at the time of discharge, when compared with those of the “normal” trajectory. Additionally, we showed that the “slow gain” trajectory was significantly associated with low GSA scores at five years of age, independently of hospital growth and others factors known to be associated with cognitive development of preterm children.

In our study, being male was found to be a significant predictor of being classified in one of the three atypical trajectories. This result is in accordance with what was previously found in regard to the BMI trajectory [8], level of prematurity [32], and associated long term adverse events [33].

It has been shown that individuals who are born preterm often exhibit a substantial growth failure in the early postnatal period, usually followed by catch-up growth over the subsequent 2 years [34]. This growth profile is typical of our group of rapid BMI Z-score gainers, and represented approximately 5% of our population. This profile was previously shown to be beneficial in terms of cognitive outcomes, but detrimental in regard to metabolic outcomes in adulthood [35]. The observed association between suboptimal initial weight gain and breastfeeding at discharge is also consistent with what has already been presented in the literature [36]. Surprisingly, associated children corresponded to those with the most severe neonatal morbidities, and therefore would have been expected to show lower GSA scores when compared to the normal group.

Several studies that focused either on children who were born preterm or who were small for their gestational age have shown that a loss of weight Z-score or a small gain in growth Z-score during early childhood is associated with a higher risk of developmental impairment later in childhood [4]. According to our LCA analysis, this population is heterogeneous, and it is composed of two distinct groups: a group of slow BMI Z-score gainers, mainly associated with low birth weight; and a group of slow BMI Z-score losers, mainly associated with both low gestational age and birth HC. These two groups represented 13% and 18% of our sample, respectively. Only the group of slow gainers was shown to be associated with cognitive impairment at 5 years of age.

All these results are an imperfect reflection of the underlying complex interplay in preterm children between prematurity, neonatal morbidities, nutrition, early neonatal and post-term growths, and long-term development. Further investigations of these aspects will be needed in order to gain a better insight regarding the key factors at play.

The current results are subject to several limitations. Firstly, to avoid a potential sensitivity of fit indices to missing measurements in regard to the BMI trajectory, we chose to include only children who had three or more BMI measurements in our analysis. When comparing children included and not included in the LCA, significant differences could be seen. Healthier preterm children were indeed overrepresented in those not included in the LCA. Absolute differences in characteristic distribution were, however, small indicating that this restriction did not result in obvious selection bias. Furthermore, this selection bias is mainly expected to affect the proportions of persons in each trajectory but is unlikely to impact the shapes of the trajectories themselves. The GSA score evaluation was also only available for 62% of the preterm children included in the LCA analysis. The rate and characteristics of attrition are similar to those of other large longitudinal studies in the areas [37] and indicated that preterm children from higher socioeconomic levels were overrepresented. Although our study was a large population-based study of 2385 children with a valid cognitive evaluation at 5 years of age, our results should hence be interpreted with a degree of caution due to this potential pitfall. A second limitation is the use of the GSA score to assess cognitive development. Although our team has established that this score is a simple and reliable screening tool for assessing cognitive development in preterm children at five years of age [26], this questionnaire was originally developed to asses a child’s abilities and behavior in the classroom. It may be influenced by subjective factors, such as the child-teacher relationship. Nonetheless, this score assesses the child in their own environment, and it compares the child with other children from the same school class who thus constitute the control group. It is of particular relevance in terms of the behavior and socialization of the children [26]. Thirdly, the main limitation is uncontrolled confounding. To control for this, we performed a propensity score analysis, and we made a rigorous adjustment for confounding factors, thereby minimizing the likelihood of incorrectly determining the association between abnormal trajectories and lower GSA scores. Data regarding perinatal or post-term characteristics that are known to influence the children’s development, such as maternal age/BMI or specific pathologies occurring during childhood are however lacking in our study. Fourthly, Z-score BMI is only one way to quantitatively describe changes of child growth over time. There is also mounting evidence that, in addition to the quantity, monitoring of the quality of growth changes may play an important role in explaining the relationship between early growth patterns and future health [38,39]. Additionally, Olsen and WHO standards were used to compute both preterm and post-term Z-scores. Other standards however exist [40]. Further studies using alternative markers of both quantity and quality of growth are needed.

## Conclusions

Our findings indicate heterogeneous post-term growth of preterm children, with potential for association with their cognitive development. Four different growth patterns were identified and a distinguished group of children characterized by a slow increase in growth during childhood was significantly associated with low cognitive development at five years of age. Additional experimental research is, however, clearly needed to confirm the observed association, so as to better understand the characteristics underlying these growth trajectories.

## Supporting information

S1 File Supplementary materials. (DOC)
