# Supplementary material for: Post-term growth and cognitive development at 5 years of age in preterm children: Evidence from a prospective population-based cohort
Source: PLoS One. 2017 Mar 28;12(3):e0174645. doi: 10.1371/journal.pone.0174645 (PMC5370142; doi:10.1371/journal.pone.0174645)
Supplement: S1 File — (DOC) [file pone.0174645.s001.doc]

# SUPPLEMENTARY MATERIALS

**Table A. Characteristics of included children according to each trajectory identified by** the latent class analysis

| **Variable** | **Category** | **Normal** | **Slow loss** | **Slow gain** | **Rapid gain** | **Total** |
| --- | --- | --- | --- | --- | --- | --- |
|  |  | **trajectory** | **trajectory** | **trajectory** | **trajectory** |  |
|  |  | **(N=2,469)** | **(N=676)** | **(N=510)** | **(N=195)** | **N=3,850** |
| ***Children’s characteristics*** | | | | | | |
| **Child’s gender, n (%)** | *Male* | 1275 (51.6) | 385 (57) | 304 (59.6) | 122 (62.6) | 2086 (54.2) |
| **Gestational age, n (%)** | *22-29* | 444 (18) | 134 (19.8) | 124 (24.3) | 44 (22.6) | 746 (19.4) |
|  | *30-31* | 445 (18) | 144 (21.3) | 95 (18.6) | 32 (16.4) | 716 (18.6) |
|  | *32-33* | 911 (36.9) | 238 (35.2) | 169 (33.1) | 69 (35.4) | 1387 (36) |
|  | *34-35* | 669 (27.1) | 160 (23.7) | 122 (23.9) | 50 (25.6) | 1001 (26) |
| **Birth weight Z-score**** | *Median (IQR)* | -0.2 (-0.9,0.4) | -0.3 (-1,0.3) | -0.6 (-1.5,0.1) | -0.1 (-0.9,0.5) | -0.3 (-1,0.4) |
| **Birth HC Z-score**** | *Median (IQR)* | -0.1 (-0.7,0.6) | -0.2 (-0.9,0.3) | -0.4 (-1,0.2) | 0 (-0.7,0.8) | -0.1 (-0.7,0.5) |
| **Discharge weight Z-score**** | *Median (IQR)* | -0.9 (-1.5,-0.3) | -0.9 (-1.6,-0.3) | -1.4 (-2,-0.7) | -1.1 (-1.8,-0.5) | -0.9 (-1.6,-0.3) |
| **Discharge height Z-score**** | *Median (IQR)* | -1.1 (-1.8,-0.5) | -1.2 (-1.9,-0.5) | -1.5 (-2.3,-0.8) | -1.4 (-2,-0.8) | -1.2 (-1.9,-0.5) |
| **Discharge HC Z-score**** | *Median (IQR)* | -0.1 (-0.6,0.4) | -0.2 (-0.7,0.4) | -0.4 (-0.9,0.1) | -0.2 (-0.9,0.2) | -0.2 (-0.7,0.4) |
|  | 2003-2004 | 693 (28.1) | 200 (29.6) | 154 (30.2) | 54 (27.7) | 1101 (28.6) |
| **Year of birth, n (%)** | 2005-2006 | 865 (35) | 227 (33.6) | 171 (33.5) | 63 (32.3) | 1326 (34.4) |
|  | 2007-2008 | 911 (36.9) | 249 (36.8) | 185 (36.3) | 78 (40) | 1423 (37) |
| ***Mother and pregnancy characteristics*** | | | | | | |
| **Socioeconomic level, n (%)** | *Higher level* | 673 (27.3) | 150 (22.2) | 127 (24.9) | 50 (25.6) | 1000 (26) |
| **Social security benefits for low income, n (%)** | Yes | 279 (11.3) | 65 (9.6) | 52 (10.2) | 24 (12.3) | 420 (10.9) |
| **Multiple pregnancy, n (%)** | Yes | 932 (37.7) | 265 (39.2) | 197 (38.6) | 62 (31.8) | 1456 (37.8) |
| **Antenatal corticotherapy, n (%)** | Yes | 1331 (53.9) | 366 (54.1) | 293 (57.5) | 104 (53.3) | 2094 (54.4) |
| **Hypertension during pregnancy, n (%)** | Yes | 344 (13.9) | 95 (14.1) | 75 (14.7) | 32 (16.4) | 546 (14.2) |
| ***Neonatal hospitalization characteristics*** | | | | | | |
| **Surgery, n (%)** | Yes | 36 (1.5) | 16 (2.4) | 17 (3.3) | 7 (3.6) | 76 (2) |
| **Severe cranial ultrasound/MRI abnormalities, n (%)** | Yes | 82 (3.3) | 27 (4) | 21 (4.1) | 9 (4.6) | 139 (3.6) |
| **Intubation at birth, n (%)** | Yes | 365 (14.8) | 114 (16.9) | 94 (18.4) | 47 (24.1) | 620 (16.1) |
| **Apgar score at 5 min, n (%)** | <7 | 102 (4.1) | 34 (5) | 30 (5.9) | 14 (7.2) | 180 (4.7) |
| **Late-onset infection, n (%)** | Yes | 311 (12.6) | 84 (12.4) | 92 (18) | 42 (21.5) | 529 (13.7) |
| **Necrotizing enterocolitis, n (%)** | Yes | 12 (0.5) | 2 (0.3) | 4 (0.8) | 4 (2.1) | 22 (0.6) |
| **Bronchopulmonary dysplasia, n (%)** | No oxygen therapy | 1642 (66.5) | 444 (65.7) | 322 (63.1) | 117 (60) | 2525 (65.6) |
|  | O2<28 days | 706 (28.6) | 198 (29.3) | 143 (28) | 59 (30.3) | 1106 (28.7) |
|  | O2 ≥ 28 days | 121 (4.9) | 34 (5) | 45 (8.8) | 19 (9.7) | 219 (5.7) |
| ***Child nutrition/growth characteristics during hospitalization*** | | | | | | |
| **Delta weight Z-score**** | *Median (IQR)* | -0.6 (-1.1,-0.2) | -0.5 (-1,-0.1) | -0.6 (-1.2,-0.1) | -0.9 (-1.5,-0.4) | -0.6 (-1.1,-0.2) |
| **Length of parenteral nutrition, n (%)** | No | 671 (27.2) | 183 (27.1) | 143 (28) | 57 (29.2) | 1054 (27.4) |
|  | < 11 days | 1153 (46.7) | 301 (44.5) | 193 (37.8) | 78 (40) | 1725 (44.8) |
|  | ≥ 11 days | 645 (26.1) | 192 (28.4) | 174 (34.1) | 60 (30.8) | 1071 (27.8) |
| **Breastfeeding at discharge, n (%)** | Yes | 441 (17.9) | 82 (12.1) | 89 (17.5) | 47 (24.1) | 659 (17.1) |
| ***Child neurodevelopment at 5 years of age (n=2385)*** | | | | | | |
| **GSA score** | *Median (IQR)* | 52 (44,56) | 52 (44,57) | 47 (38,55) | 51 (43,56) | 51 (43,56) |

IQR: interquartile range

BMI: Body Mass Index

HC: Head Circumference

** Z-scores were computed according to Olsen’ standards

**Table B. Post-term anthropometric data of included children at 3, 9, 18, and 24 months and 3, 4, and 5 years of post-menstrual age according to each trajectory identified by** the latent class analysis

|  | **Normal** | **Slow loss** | **Slow gain** | **Rapid gain** | **Total** |
| --- | --- | --- | --- | --- | --- |
|  | **trajectory** | **trajectory** | **trajectory** | **trajectory** |
|  | **(N=2,469)** | **(N=676)** | **(N=510)** | **(N=195)** | **(N=3,850)** |
|  | **Median [IQR]** | **Median [IQR]** | **Median [IQR]** | **Median [IQR]** | **Median [IQR]** |
| **3-month visit** | | | | | |
| **Weight Z-score*** | -0.5 [-1.2; 0.2] | -0.3 [-0.9; 0.4] | -1.6 [-2.5; -0.9] | -2 [-3; -0.9] | -0.6 [-1.4; 0.1] |
| **Height Z-score*** | -0.5 [-1.4; 0.2] | -0.8 [-1.6; 0] | -1 [-2; -0.2] | -1.1 [-2.1; 0.1] | -0.6 [-1.6; 0.1] |
| **HC Z-Score*** | 0.5 [-0.2; 1.2] | 0.3 [-0.3; 1.1] | 0 [-0.7; 0.7] | 0 [-1; 0.8] | 0.4 [-0.4; 1.1] |
| **BMI Z-score*** | -0.3 [-0.9; 0.3] | 0.2 [-0.4; 0.9] | -1.4 [-2; -0.8] | -2.1 [-2.8; -1.4] | -0.4 [-1.1; 0.3] |
| **9-month visit** | | | | | |
| **Weight Z-score*** | 0.0 [-0.7; 0.7] | -0.4 [-1.1; 0.3] | -1.3 [-2; -0.6] | -0.8 [-1.6; 0.1] | -0.3 [-1; 0.5] |
| **Height Z-score*** | -0.1 [-0.9; 0.6] | -0.3 [-1.0; 0.4] | -0.4 [-1.3; 0.5] | -0.5 [-1.5; 0.3] | -0.2 [-1; 0.5] |
| **HC Z-Score*** | 0.8 [0.0; 1.5] | 0.5 [-0.2; 1.2] | 0.1 [-0.5; 1.1] | 0.5 [-0.4; 1.5] | 0.7 [-0.1; 1.5] |
| **BMI Z-score*** | 0.0 [-0.5; 0.7] | -0.3 [-0.9; 0.4] | -1.5 [-2; -0.9] | -0.5 [-1.5; 0.2] | -0.2 [-0.9; 0.5] |
| **18-month visit** | | | | | |
| **Weight Z-score*** | 0 [-0.6; 0.6] | -0.6 [-1.2; -0.1] | -0.9 [-1.7; -0.3] | 0 [-0.8; 0.6] | -0.2 [-0.9; 0.4] |
| **Height Z-score*** | -0.2 [-0.9; 0.5] | -0.3 [-1; 0.3] | -0.4 [-1.3; 0.4] | -0.6 [-1.4; 0.2] | -0.3 [-1; 0.5] |
| **HC Z-Score*** | 0.8 [0; 1.5] | 0.4 [-0.3; 1.2] | 0.2 [-0.5; 1.2] | 0.6 [-0.3; 1.8] | 0.5 [-0.2; 1.3] |
| **BMI Z-score*** | 0.2 [-0.3; 0.8] | -0.7 [-1.2; -0.1] | -1 [-1.6; -0.5] | 0.4 [-0.3; 1.1] | -0.1 [-0.7; 0.6] |
| **24-month visit** | | | | | |
| **Weight Z-score*** | 0.0 [-0.6; 0.6] | -0.7 [-1.4; -0.2] | -0.8 [-1.6; -0.2] | 0.1 [-0.5; 0.8] | -0.2 [-0.9; 0.4] |
| **Height Z-score*** | 0.0 [-0.7; 0.7] | -0.1 [-0.8; 0.5] | -0.2 [-1; 0.5] | -0.3 [-1; 0.4] | -0.1 [-0.8; 0.6] |
| **HC Z-Score*** | 0.8 [0.1; 1.5] | 0.4 [-0.4; 1.1] | 0.3 [-0.5; 1.2] | 1.1 [-0.2; 1.9] | 0.6 [-0.2; 1.3] |
| **BMI Z-score*** | 0.0 [-0.6; 0.6] | -1.1 [-1.7; -0.5] | -1.1 [-1.7; -0.5] | 0.3 [-0.3; 1.2] | -0.3 [-1; 0.3] |
| **36-month visit** | | | | | |
| **Weight Z-score*** | -0.1 [-0.7; 0.5] | -0.8 [-1.4; -0.3] | -0.6 [-1.4; 0] | 0.1 [-0.6; 0.7] | -0.3 [-1; 0.3] |
| **Height Z-score*** | -0.1 [-0.7; 0.6] | -0.3 [-0.9; 0.4] | -0.2 [-1.1; 0.4] | -0.3 [-0.9; 0.5] | -0.1 [-0.8; 0.5] |
| **HC Z-Score*** | 0.5 [-0.2; 1.4] | 0.1 [-0.6; 0.8] | 0.4 [-0.6; 1.1] | 0.5 [-0.2; 1.3] | 0.5 [-0.3; 1.2] |
| **BMI Z-score*** | -0.2 [-0.8; 0.4] | -1.1 [-1.7; -0.5] | -0.8 [-1.4; -0.1] | 0.4 [-0.4; 0.9] | -0.4 [-1.1; 0.3] |
| **48-month visit** | | | | | |
| **Weight Z-score*** | -0.2 [-0.8; 0.4] | -0.7 [-1.3; -0.1] | -0.4 [-1.1; 0.3] | 0.1 [-0.5; 0.6] | -0.3 [-0.9; 0.3] |
| **Height Z-score*** | -0.1 [-0.7; 0.5] | -0.4 [-0.9; 0.3] | -0.3 [-1; 0.4] | 0 [-0.6; 0.6] | -0.2 [-0.8; 0.5] |
| **HC Z-Score*** | 0.5 [-0.2; 1.2] | -0.1 [-0.8; 0.8] | 0.3 [-0.6; 1] | 0.6 [-0.1; 1.5] | 0.5 [-0.2; 1.2] |
| **BMI Z-score*** | -0.2 [-0.8; 0.4] | -0.8 [-1.5; -0.3] | -0.4 [-1; 0.3] | 0.2 [-0.7; 0.7] | -0.3 [-1; 0.3] |
| **60-month visit** | | | | | |
| **Weight Z-score*** | -0.2 [-0.8; 0.4] | -0.6 [-1.1; -0.1] | -0.2 [-1; 0.6] | 0.1 [-0.6; 0.6] | -0.2 [-0.9; 0.4] |
| **Height Z-score*** | -0.1 [-0.7; 0.6] | -0.3 [-0.9; 0.3] | -0.1 [-0.9; 0.5] | -0.2 [-0.6; 0.6] | -0.1 [-0.8; 0.5] |
| **HC Z-Score*** | 0.4 [-0.3; 1.2] | 0.1 [-0.6; 0.8] | 0.2 [-0.6; 0.9] | 0.5 [-0.5; 1.2] | 0.2 [-0.5; 1.1] |
| **BMI Z-score*** | -0.2 [-0.8; 0.4] | -0.7 [-1.3; -0.1] | -0.2 [-0.9; 0.5] | 0.1 [-0.8; 0.8] | -0.3 [-0.9; 0.4] |

IQR: interquartile range; BMI: Body Mass Index; HC: Head Circumference

* Z-scores were computed according to the ‘‘WHO child growth standards’’ for children less than 1,856 days of age, and according to the ‘‘WHO growth standards for school-aged children and adolescents’’ thereafter

**Table C. BIC criteria and smallest class size for the estimated latent class models**

| **Number of trajectories** | **BIC** | **Smallest class size** |
| --- | --- | --- |
| 2 | 50914.13 | 451 (11.71%) |
| 3 | 50719.48 | 216 (5.61%) |
| 4 | 50509.69 | 195 (5.06%) |
| 5 | 50443.57 | 19 (0.49%) |

**Table D. Estimates of the final latent class model in regard to 4 longitudinal BMI z-score trajectories in children from the LIFT cohort (N=5,458) (Time unit: year)**

|  |  | **Estimate** | **Standard error** |
| --- | --- | --- | --- |
| **Trajectory 1** | *Intercept* | 0.38229 | 0.10556 |
|  | *Time* | -0.87703 | 0.17960 |
|  | *Time ^2^* | 0.18056 | 0.06507 |
|  | *Time ^3^* | -0.00850 | 0.00691 |
| **Trajectory 2** | *Intercept* | -1.23635 | 0.09271 |
|  | *Time* | 0.27861 | 0.11556 |
|  | *Time ^2^* | -0.02702 | 0.05506 |
|  | *Time ^3^* | 0.00283 | 0.00673 |
| **Trajectory 3** | *Intercept* | -0.40582 | 0.05737 |
|  | *Time* | 0.85166 | 0.11091 |
|  | *Time ^2^* | -0.39304 | 0.04066 |
|  | *Time ^3^* | 0.04636 | 0.00428 |
| **Trajectory 4** | *Intercept* | -2.71683 | 0.15839 |
|  | *Time* | 3.67969 | 0.17833 |
|  | *Time ^2^* | -1.27747 | 0.07398 |
|  | *Time ^3^* | 0.13077 | 0.00896 |

**Figure A. Mean weight, height and head circumference Z-scores between 3 and 60 months in children from the four distinct BMI Z-score trajectories identified by the final latent class model. Z-scores were computed according to the ‘‘WHO child growth standards’’ for children less than 1,856 days of age, and according to the ‘‘WHO growth standards for school-aged children and adolescents’’ thereafter.**

**
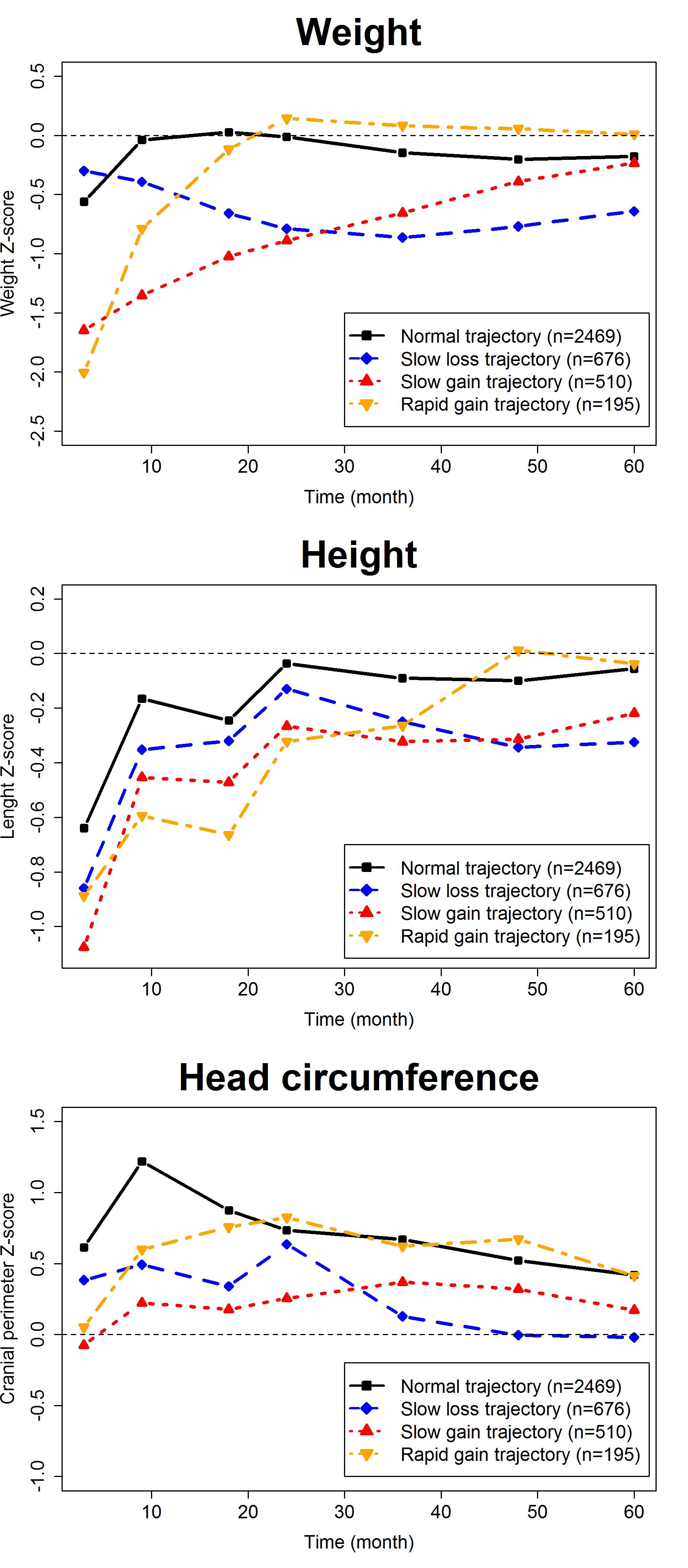
**

**Figure B. Mean weight Z-scores between 3 and 60 months in children from the four distinct BMI Z-score trajectories identified by the final latent class model when only one child from those of a same multiple pregnancy is included in the LCA. Z-scores were computed according to the ‘‘WHO child growth standards’’ for children less than 1,856 days of age, and according to the ‘‘WHO growth standards for school-aged children and adolescents’’ thereafter.**

**
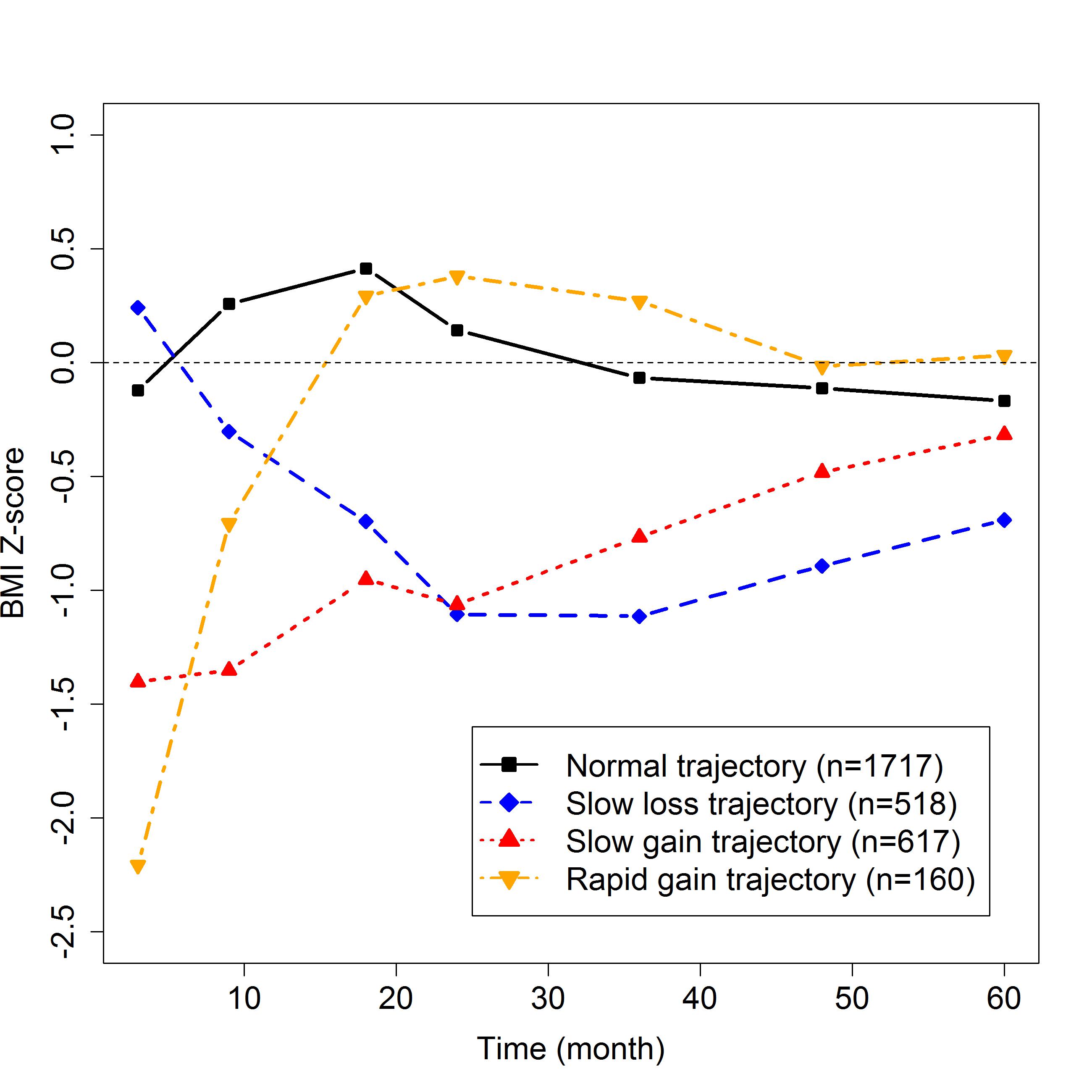
**

**Table E. Baseline characteristics in the overall cohort (N=1,932) and in the matched cohort (N = 1,613) used for the GSA analysis comparing the “normal” and “slow loss” BMI Z-score trajectories**

| **Variable** | **Category** | **Overall Cohort** | | | **Matched Cohort** | | |
| --- | --- | --- | --- | --- | --- | --- | --- |
|  |  | **Normal trajectory** | **Slow loss trajectory** | **Standardized** | **Normal trajectory** | **Slow loss trajectory** | **Standardized** |
|  |  | ***N = 1,509*** | ***N = 423*** | **difference (%)** | **N = 1,191** | **N = 422** | **difference (%)** |
| **Children’s characteristics** | | | | | | | |
| **Child’s gender** | *Male* | 787 (52.2) | 238 (56.3) | 8.2 | 645 (54.2) | 237 (56.2) | 4 |
| **Gestational age** | *22-29* | 267 (17.7) | 91 (21.5) | 9.8 | 236 (19.8) | 90 (21.3) | 3.8 |
|  | *30-31* | 273 (18.1) | 79 (18.7) | 1.5 | 217 (18.2) | 79 (18.7) | 1.3 |
|  | *32-33* | 564 (37.4) | 161 (38.1) | 1.4 | 450 (37.8) | 161 (38.2) | 0.8 |
|  | *34-35* | 405 (26.8) | 92 (21.7) | 11.6 | 288 (24.2) | 92 (21.8) | 5.6 |
| **Birth weight Z-score*** | *Median (IQR)* | -0.24 (1) | -0.39 (1) | 15.4 | -0.36 (1) | -0.39 (1) | 2.5 |
| **Birth HC Z-score*** | *Median (IQR)* | -0.04 (1) | -0.21 (0.9) | 18 | -0.15 (1) | -0.21 (0.9) | 6.2 |
| **Discharge weight Z-score*** | *Median (IQR)* | -0.89 (1.4) | -0.91 (1.4) | 1.8 | -0.97 (1.3) | -0.91 (1.4) | 4.3 |
| **Discharge height Z-score*** | *Median (IQR)* | 46.18 (3.4) | 46.02 (3.5) | 4.7 | 45.98 (3.5) | 46.03 (3.4) | 1.6 |
| **Discharge HC Z-score*** | *Median (IQR)* | -0.07 (0.9) | -0.2 (0.9) | 15.2 | -0.12 (0.8) | -0.2 (0.9) | 8.8 |
| **Year of birth** | 2003-2004 | 415 (27.5) | 135 (31.9) | 9.8 | 317 (26.6) | 135 (32) | 12 |
|  | 2005-2006 | 523 (34.7) | 129 (30.5) | 8.8 | 416 (34.9) | 129 (30.6) | 9.2 |
|  | 2007-2008 | 571 (37.8) | 159 (37.6) | 0.5 | 458 (38.5) | 158 (37.4) | 2.1 |
| **Mother and pregnancy characteristics** | | | | | | | |
| **Socioeconomic level** | *Higher level* | 463 (30.7) | 101 (23.9) | 15 | 330 (27.7) | 101 (23.9) | 8.5 |
| **Social security benefits for low income** | Yes | 143 (9.5) | 38 (9) | 1.7 | 111 (9.3) | 38 (9) | 1.1 |
| **Multiple pregnancy** | Yes | 577 (38.2) | 155 (36.6) | 3.3 | 464 (39) | 155 (36.7) | 4.6 |
| **Antenatal corticotherapy** | Yes | 816 (54.1) | 237 (56) | 3.9 | 644 (54.1) | 237 (56.2) | 4.2 |
| **Hypertension during pregnancy** | Yes | 206 (13.7) | 62 (14.7) | 2.9 | 166 (13.9) | 62 (14.7) | 2.2 |
| ***Neonatal hospitalization characteristics*** | | | | | | | |
| **Surgery** | Yes | 27 (1.8) | 12 (2.8) | 7.4 | 26 (2.2) | 12 (2.8) | 4.4 |
| **Severe cranial ultrasound/MRI abnormalities** | Yes | 46 (3) | 18 (4.3) | 6.7 | 44 (3.7) | 18 (4.3) | 3 |
| **Intubation at birth** | Yes | 221 (14.6) | 75 (17.7) | 8.6 | 194 (16.3) | 74 (17.5) | 3.3 |
| **Apgar at 5 min** | <7 | 58 (3.8) | 20 (4.7) | 4.5 | 52 (4.4) | 20 (4.7) | 1.8 |
| **Late-onset infection** | Yes | 182 (12.1) | 59 (13.9) | 5.7 | 149 (12.5) | 58 (13.7) | 3.7 |
| **Necrotizing enterocolitis** | Yes | 6 (0.4) | 1 (0.2) | 2.7 | 3 (0.3) | 1 (0.2) | 0.3 |
| **Bronchopulmonary dysplasia** | No oxygen therapy | 1003 (66.5) | 277 (65.5) | 2.1 | 777 (65.2) | 276 (65.4) | 0.3 |
|  | O2<28 days | 434 (28.8) | 123 (29.1) | 0.7 | 350 (29.4) | 123 (29.1) | 0.5 |
|  | O2 ≥ 28 days | 72 (4.8) | 23 (5.4) | 3.1 | 64 (5.4) | 23 (5.5) | 0.3 |
| ***Child nutrition/growth characteristics during hospitalization*** | | | | | | | |
| **Delta weight Z-score*** | *Median (IQR)* | -0.67 (0.9) | -0.55 (1) | 12.1 | -0.61 (0.9) | -0.56 (1) | 5.8 |
| **Length of parenteral nutrition** | No | 405 (26.8) | 120 (28.4) | 3.4 | 315 (26.4) | 119 (28.2) | 3.9 |
|  | < 11 days | 706 (46.8) | 185 (43.7) | 6.1 | 540 (45.3) | 185 (43.8) | 3 |
|  | ≥ 11 days | 398 (26.4) | 118 (27.9) | 3.4 | 336 (28.2) | 118 (28) | 0.6 |
| **Breast feeding at discharge** | Yes | 286 (19) | 54 (12.8) | 16.2 | 184 (15.4) | 54 (12.8) | 7.5 |

BMI: Body Mass Index

HC: Head Circumference

**Table F. Baseline characteristics in the overall cohort (N=1,840) and in the matched cohort (N = 1232) used for the GSA analysis comparing the “normal” and “slow gain” BMI Z-score trajectories**

| **Variable** | **Category** | **Overall Cohort** | | | **Matched Cohort** | | |
| --- | --- | --- | --- | --- | --- | --- | --- |
|  |  | **Normal trajectory** | **Slow gain trajectory** | **Standardized** | **Normal trajectory** | **Slow gain trajectory** | **Standardized** |
|  |  | ***N = 1,509*** | ***N = 331*** | **difference (%)** | **N = 906** | **N = 326** | **difference (%)** |
| **Children’s characteristics** | | | | | | | |
| **Child’s gender** | *Male* | 787 (52.2) | 197 (59.5) | 14.8 | 509 (56.2) | 193 (59.2) | 6.1 |
| **Gestational age** | *22-29* | 267 (17.7) | 77 (23.3) | 14.3 | 189 (20.9) | 75 (23) | 5.2 |
|  | *30-31* | 273 (18.1) | 65 (19.6) | 4 | 177 (19.5) | 63 (19.3) | 0.5 |
|  | *32-33* | 564 (37.4) | 102 (30.8) | 13.7 | 284 (31.3) | 101 (31) | 0.8 |
|  | *34-35* | 405 (26.8) | 87 (26.3) | 1.3 | 256 (28.3) | 87 (26.7) | 3.5 |
| **Birth weight Z-score** | *Median (IQR)* | -0.24 (1) | -0.69 (1.1) | 43.3 | -0.59 (1) | -0.66 (1) | 6.8 |
| **Birth HC Z-score** | *Median (IQR)* | -0.04 (1) | -0.4 (1) | 36.5 | -0.25 (1) | -0.38 (1) | 13.4 |
| **Discharge weight Z-score** | *Median (IQR)* | -0.89 (1.4) | -1.35 (1.1) | 32.9 | -1.22 (1.1) | -1.32 (1.1) | 9.4 |
| **Discharge height Z-score** | *Median (IQR)* | -1.14 (1) | -1.52 (1.1) | 36.5 | -1.43 (1) | -1.5 (1.1) | 6.5 |
| **Discharge HC Z-score** | *Median (IQR)* | -0.07 (0.9) | -0.37 (0.8) | 34.4 | -0.26 (0.9) | -0.35 (0.8) | 11 |
| **Year of birth** | 2003-2004 | 415 (27.5) | 95 (28.7) | 2.7 | 258 (28.5) | 93 (28.5) | 0.1 |
|  | 2005-2006 | 523 (34.7) | 101 (30.5) | 8.8 | 317 (35) | 99 (30.4) | 9.8 |
|  | 2007-2008 | 571 (37.8) | 135 (40.8) | 6.1 | 331 (36.5) | 134 (41.1) | 9.4 |
| **Mother and pregnancy characteristics** | | | | | | | |
| **Socioeconomic level** | *Higher level* | 463 (30.7) | 89 (26.9) | 8.3 | 265 (29.2) | 86 (26.4) | 6.4 |
| **Social security benefits for low income** | Yes | 143 (9.5) | 35 (10.6) | 3.7 | 88 (9.7) | 35 (10.7) | 3.4 |
| **Multiple pregnancy** | Yes | 577 (38.2) | 137 (41.4) | 6.5 | 351 (38.7) | 133 (40.8) | 4.2 |
| **Antenatal corticotherapy** | Yes | 816 (54.1) | 178 (53.8) | 0.6 | 511 (56.4) | 173 (53.1) | 6.7 |
| **Hypertension during pregnancy** | Yes | 206 (13.7) | 49 (14.8) | 3.3 | 117 (12.9) | 49 (15) | 6.2 |
| ***Neonatal hospitalization characteristics*** | | | | | | | |
| **Surgery** | Yes | 27 (1.8) | 7 (2.1) | 2.4 | 19 (2.1) | 6 (1.8) | 1.8 |
| **Severe cranial ultrasound/MRI abnormalities** | Yes | 46 (3) | 13 (3.9) | 5 | 33 (3.6) | 13 (4) | 1.8 |
| **Intubation at birth** | Yes | 221 (14.6) | 62 (18.7) | 11.3 | 150 (16.6) | 60 (18.4) | 4.9 |
| **Apgar at 5 min** | <7 | 58 (3.8) | 23 (6.9) | 15.1 | 37 (4.1) | 22 (6.7) | 12.5 |
| **Late-onset infection** | Yes | 182 (12.1) | 56 (16.9) | 14.5 | 129 (14.2) | 53 (16.3) | 5.7 |
| **Necrotizing enterocolitis** | Yes | 6 (0.4) | 1 (0.3) | 1.6 | 4 (0.4) | 1 (0.3) | 2.1 |
| **Bronchopulmonary dysplasia** | No oxygen therapy | 1003 (66.5) | 210 (63.4) | 6.4 | 601 (66.3) | 208 (63.8) | 5.3 |
|  | O2<28 days | 434 (28.8) | 95 (28.7) | 0.1 | 248 (27.4) | 94 (28.8) | 3.3 |
|  | O2 ≥ 28 days | 72 (4.8) | 26 (7.9) | 13.7 | 57 (6.3) | 24 (7.4) | 4.3 |
| ***Child nutrition/growth characteristics during hospitalization*** | | | | | | | |
| **Delta weight Z-score** | *Median (IQR)* | -0.67 (0.9) | -0.65 (0.9) | 1.9 | -0.59 (0.9) | -0.65 (0.9) | 6.9 |
| **Length of parenteral nutrition** | No | 405 (26.8) | 99 (29.9) | 6.9 | 237 (26.2) | 98 (30.1) | 8.8 |
|  | < 11 days | 706 (46.8) | 124 (37.5) | 18.7 | 385 (42.5) | 124 (38) | 9.1 |
|  | ≥ 11 days | 398 (26.4) | 108 (32.6) | 14 | 284 (31.3) | 104 (31.9) | 1.2 |
| **Breast feeding at discharge** | Yes | 286 (19) | 69 (20.8) | 4.8 | 163 (18) | 69 (21.2) | 8.1 |

BMI: Body Mass Index

HC: Head Circumference

**Table G. Baseline characteristics in the overall cohort (N=1,631) and in the matched cohort (N = 448) used for the GSA analysis comparing the “normal” and “rapid gain” BMI Z-score trajectories**

| **Variable** | **Category** | **Overall Cohort** | | | **Matched Cohort** | | |
| --- | --- | --- | --- | --- | --- | --- | --- |
|  |  | **Normal trajectory** | **Rapid gain trajectory** | **Standardized** | **Normal trajectory** | **Rapid gain trajectory** | **Standardized** |
|  |  | ***N = 1509*** | ***N = 122*** | **difference (%)** | **N = 333** | **N = 115** | **difference (%)** |
| **Children’s characteristics** | | | | | | | |
| **Child’s gender** | *Male* | 787 (52.2) | 75 (61.5) | 18.7 | 199 (59.8) | 70 (60.9) | 2.3 |
| **Gestational age** | *22-29* | 267 (17.7) | 28 (23) | 13.7 | 76 (22.8) | 25 (21.7) | 2.6 |
|  | *30-31* | 273 (18.1) | 14 (11.5) | 17.4 | 36 (10.8) | 13 (11.3) | 1.6 |
|  | *32-33* | 564 (37.4) | 48 (39.3) | 4.1 | 139 (41.7) | 45 (39.1) | 5.3 |
|  | *34-35* | 405 (26.8) | 32 (26.2) | 1.4 | 82 (24.6) | 32 (27.8) | 7.4 |
| **Birth weight Z-score** | *Median (IQR)* | -0.24 (1) | -0.11 (1.1) | 12.9 | -0.13 (1.1) | -0.17 (1.1) | 3.5 |
| **Birth HC Z-score** | *Median (IQR)* | -0.04 (1) | 0.05 (1) | 9.1 | 0.03 (1) | 0.02 (1) | 1 |
| **Discharge weight Z-score** | *Median (IQR)* | -0.89 (1.4) | -1.09 (0.9) | 14.3 | -0.9 (1.7) | -1.08 (0.9) | 11.5 |
| **Discharge height Z-score** | *Median (IQR)* | -1.14 (1) | -1.32 (1.1) | 17.7 | -1.18 (1) | -1.32 (1.1) | 13.6 |
| **Discharge HC Z-score** | *Median (IQR)* | -0.07 (0.9) | -0.19 (0.8) | 14.2 | -0.18 (0.8) | -0.21 (0.8) | 3.9 |
| **Year of birth** | 2003-2004 | 415 (27.5) | 36 (29.5) | 4.5 | 85 (25.5) | 35 (30.4) | 11.1 |
|  | 2005-2006 | 523 (34.7) | 36 (29.5) | 10.9 | 126 (37.8) | 36 (31.3) | 13.6 |
|  | 2007-2008 | 571 (37.8) | 50 (41) | 6.5 | 122 (36.6) | 44 (38.3) | 3.4 |
| **Mother and pregnancy characteristics** | | | | | | | |
| **Socioeconomic level** | *Higher level* | 463 (30.7) | 36 (29.5) | 2.5 | 87 (26.1) | 34 (29.6) | 7.7 |
| **Social security benefits for low income** | Yes | 143 (9.5) | 13 (10.7) | 4 | 33 (9.9) | 10 (8.7) | 4.1 |
| **Multiple pregnancy** | Yes | 577 (38.2) | 39 (32) | 12.9 | 104 (31.2) | 39 (33.9) | 5.8 |
| **Antenatal corticotherapy** | Yes | 816 (54.1) | 66 (54.1) | 0 | 170 (51.1) | 61 (53) | 4 |
| **Hypertension during pregnancy** | Yes | 206 (13.7) | 21 (17.2) | 10.3 | 39 (11.7) | 20 (17.4) | 16.8 |
| ***Neonatal hospitalization characteristics*** | | | | | | | |
| **Surgery** | Yes | 27 (1.8) | 3 (2.5) | 5 | 11 (3.3) | 3 (2.6) | 4 |
| **Severe cranial ultrasound/MRI abnormalities** | Yes | 46 (3) | 6 (4.9) | 10.6 | 13 (3.9) | 3 (2.6) | 7 |
| **Intubation at birth** | Yes | 221 (14.6) | 28 (23) | 23.1 | 69 (20.7) | 22 (19.1) | 4 |
| **Apgar at 5 min** | <7 | 58 (3.8) | 10 (8.2) | 21.8 | 23 (6.9) | 7 (6.1) | 3.3 |
| **Late-onset infection** | Yes | 182 (12.1) | 27 (22.1) | 30.1 | 56 (16.8) | 20 (17.4) | 1.5 |
| **Necrotizing enterocolitis** | Yes | 6 (0.4) | 3 (2.5) | 27.8 | 4 (1.2) | 0 (0) | 12.8 |
| **Bronchopulmonary dysplasia** | No oxygen therapy | 1003 (66.5) | 73 (59.8) | 14 | 215 (64.6) | 69 (60) | 9.5 |
|  | O2<28 days | 434 (28.8) | 39 (32) | 7.1 | 90 (27) | 37 (32.2) | 11.4 |
|  | O2 ≥ 28 days | 72 (4.8) | 10 (8.2) | 15.7 | 28 (8.4) | 9 (7.8) | 2.1 |
| ***Child nutrition/growth characteristics during hospitalization*** | | | | | | | |
| **Delta weight Z-score** | *Median (IQR)* | -0.67 (0.9) | -0.96 (0.8) | 33.1 | -0.82 (0.9) | -0.89 (0.8) | 8 |
| **Length of parenteral nutrition** | No | 405 (26.8) | 36 (29.5) | 6 | 91 (27.3) | 35 (30.4) | 6.9 |
|  | < 11 days | 706 (46.8) | 52 (42.6) | 8.3 | 145 (43.5) | 51 (44.3) | 1.6 |
|  | ≥ 11 days | 398 (26.4) | 34 (27.9) | 3.4 | 97 (29.1) | 29 (25.2) | 8.7 |
| **Breast feeding at discharge** | Yes | 286 (19) | 37 (30.3) | 28.5 | 84 (25.2) | 33 (28.7) | 7.9 |

BMI: Body Mass Index

HC: Head Circumference

* Z-scores were computed according to Olsen’ standards

**Figure C. Association between abnormal BMI Z-score trajectories and low GSA scores at five years of age when only one child from those of a same multiple pregnancy is included in the LCA using logistic regressions adjusted for gender, birth weight Z-score, and gestational age and based on propensity score matching. The reference trajectory is the “normal” trajectory.**

**
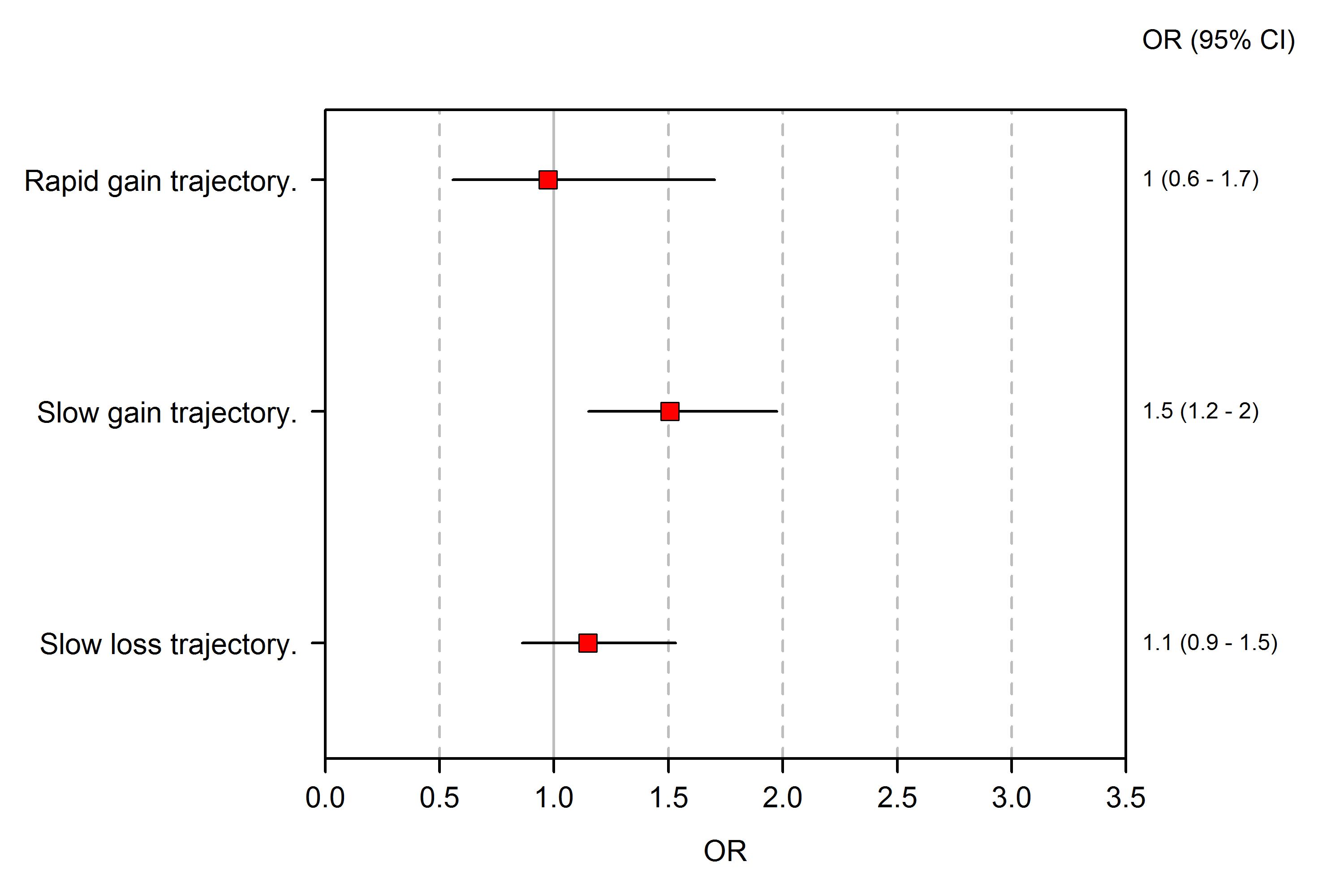
**

Propensity score matching were performed using a set of 20 variables reflecting the characteristics of the child, the characteristics of the mother and her pregnancy, the characteristics of the neonatal hospitalization, as well as the child nutrition/growth characteristics during hospitalization.

**Figure D. Association between abnormal BMI Z-score trajectories and low GSA scores at five years of age using logistic regressions adjusted for gender, birth weight Z-score, gestational age and hospital growth and based on propensity score matching. The reference trajectory is the “normal” trajectory.**

**
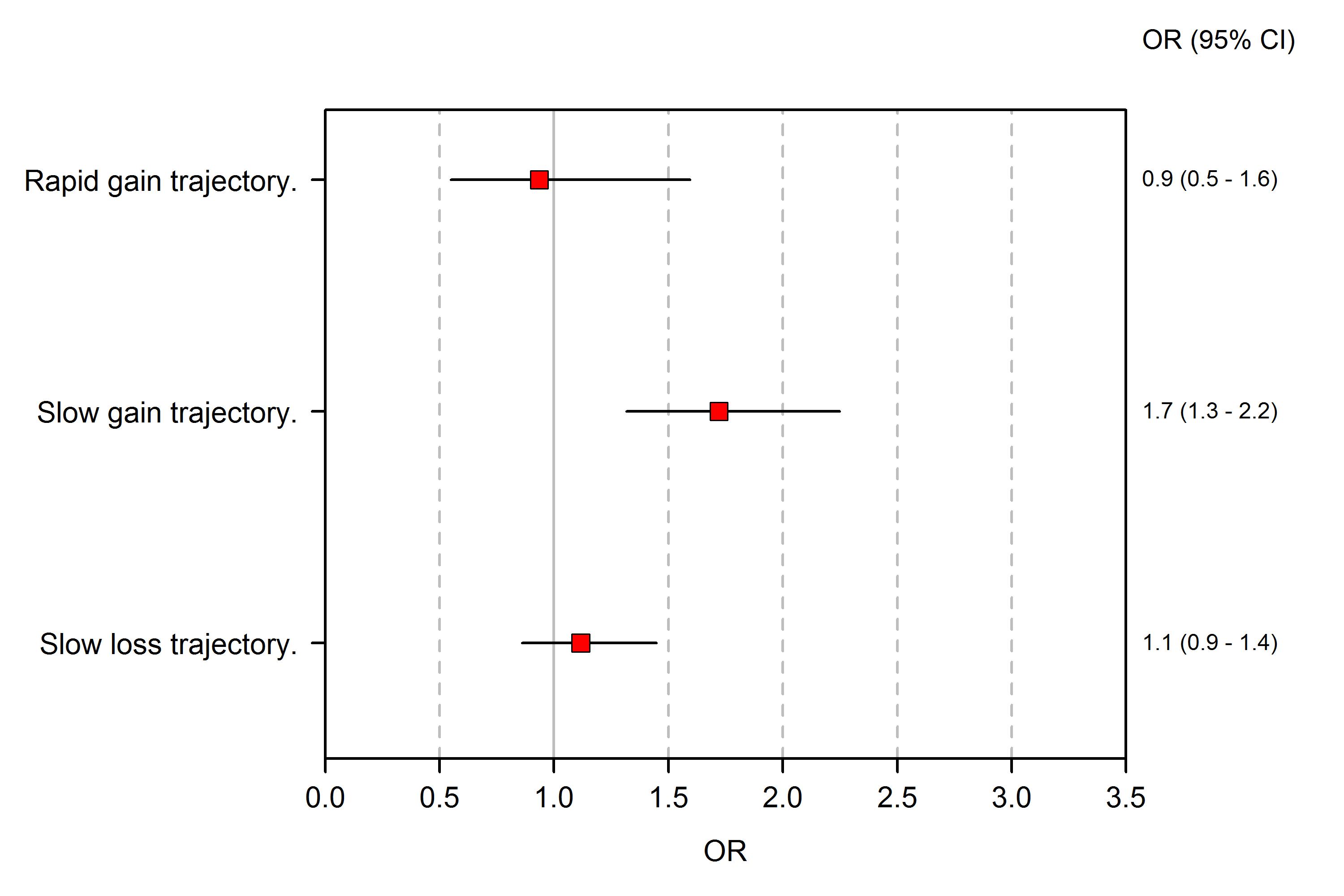
**

Propensity score matching were performed using a set of 20 variables reflecting the characteristics of the child, the characteristics of the mother and her pregnancy, the characteristics of the neonatal hospitalization, as well as the child nutrition/growth characteristics during hospitalization.
